# Composition, Functionality, and Use of Plantain Peel (*Musa paradisiaca*): A Scoping Review

**DOI:** 10.3390/foods15071133

**Published:** 2026-03-25

**Authors:** Andrea Pissatto Peres, Cláudia Puerari, Bruna Teles Soares Beserra, Juliana Aparecida Correia Bento, Maressa Caldeira Morzelle, Giuseppe Zeppa

**Affiliations:** 1 Department of Food and Nutrition, Faculty of Nutrition, Federal University of Mato Grosso, Cuiabá 78060-900, MG, Brazilbrunna.tsb@gmail.com (B.T.S.B.);; 2 Department of Agricultural, Forest and Food Sciences (DISAFA), University of Turin, 10095 Grugliasco, TO, Italy

**Keywords:** bioactive compounds, *Musa paradisiaca*, chemical composition, waste, antimicrobial

## Abstract

Plantain (*Musa paradisiaca*) peel is an agro-industrial waste product with remarkable functional potential, attributed to its composition of bioactive compounds with antioxidant and antimicrobial properties. Given this scenario, this scoping review aimed to map and synthesize the scientific evidence regarding the nutritional composition and potential functionalities of plantain peel. A scoping review approach was used, and data were reported using the PRISMA-ScR checklist. The studies evaluating the use of plantain peel were included without restrictions on language or publication date. The following databases were searched: Embase, MEDLINE (via PubMed), Scopus, and Web of Science. Additional searches were conducted through Google Scholar. The protocol has been registered prospectively on the Open Science Framework. This review’s findings included 53 studies. All of them presented methodological limitations that hindered further analysis and the generation of robust evidence. This analysis detailed the chemical composition of the peel, showing that it varies with ripeness stage and processing and is an excellent source of fiber and minerals. Several technological applications are explored, including the use of peel in the production of functional foods, the development of nanoparticles with antimicrobial activity, and its use as a substrate for the biosynthesis of industrial enzymes and citric acid. This review also addresses the possible health benefits that have already been studied in animal and in vitro models. Plantain peel is a promising agro-industrial by-product with high fiber, starch, and bioactive compound content and functional properties. Despite advances, challenges in sensory acceptance and process standardization limit industrial application. A key research gap remains in the systematic evaluation of antinutrient reduction (e.g., oxalates, phytates) and pesticide residue levels during the processing of plantain peel, a mandatory step before its widespread application in the food industry (e.g., flours and food additives). Further research on optimization and bioactive mechanisms is essential to enable its large-scale use and strengthen its role in the circular bioeconomy and human health.

## 1. Introduction

Plantain (*Musa paradisiaca*) is the fruit of an herbaceous plant, whose true stem is a thick, succulent underground rhizome. The pseudostem, commonly mistaken for a trunk, is formed by the bases above the leaves, which are large, light green in color, and shiny in appearance. The inflorescence appears in the form of a bunch, originating from the “heart” of the plantain tree, the structure that gives rise to the flowers [1,2].

It is crucial to distinguish between plantains (cooking bananas) and dessert bananas within the *Musa* genus to interpret the functionality of their by-products correctly. While taxonomic classification has evolved to describe most cultivated varieties as hybrids of *Musa acuminata* and *Musa balbisiana*, the binomial *Musa paradisiaca* L. is traditionally used in the literature to designate the AAB genomic group, specifically, the starchy plantains intended for cooking. This review focuses strictly on *Musa paradisiaca* (plantain) peels, as their physicochemical profile differs significantly from that of dessert bananas (often *M. acuminata* or *M. sapientum*). Plantain peels are characterized by a considerably higher starch-to-sugar ratio, lower moisture content, and distinct phenolic profiles [3]. These factors directly dictate their suitability for specific technological applications such as flour production, starch isolation, and fermentation substrates [4,5].

During industrial or domestic processing of fruit to produce juices, wines, jams, preserves, and chips, among others, substantial volumes of organic waste are generated. Traditionally treated as waste, these materials, often referred to as co-products or by-products, have an interesting chemical composition, which gives them high potential for reuse in the production chain. Research on these by-products is essential for developing sustainable recovery strategies aligned with the principles of the circular economy and the bioeconomy, by promoting the full use of resources, reducing losses, and adding value to waste [6].

The scientific pursuit of valorizing plantain peel is historically grounded in its use in traditional and folk medicine across tropical regions, especially in Africa and Asia. Historically considered a waste product for most commercial operations, the peel has long been employed by traditional practitioners for its therapeutic properties. Ethnobotanical uses often revolve around its astringent, anti-inflammatory, and wound-healing properties, including the application of peel ash or paste for treating burns, wounds, minor skin infections, ulcers, and gastrointestinal ailments such as diarrhea and dysentery. The current scientific investigation into the peel’s functionality, particularly its antioxidant, antimicrobial, and antidiabetic potential, aims to validate and systematically explore the biochemical basis for these traditional applications, transforming a historical waste material into a novel resource for the food and pharmaceutical industries [7,8].

Specifically, plantain peel has attracted scientific and industrial interest due to its rich composition, which possess antioxidant and anti-inflammatory properties. Advances in scientific research have revealed a variety of promising applications, including its use in the formulation of functional foods, in the production of enzymes, in the development of edible biofilms, and as a natural preservative. In addition, studies have highlighted the therapeutic potential of the peel, suggesting its application in the prevention and treatment of certain diseases [7,9,10,11,12].

Therefore, this scoping review aimed to map and synthesize the evidence available in the scientific literature on the nutritional composition and potential functionalities of plantain peel. Identifying this knowledge is necessary to expand the applicability of plantain peel in different contexts, including enzyme production, microbiological control, the development of new food products, and the incorporation into formulations of breads, cookies, pasta, jams, fermented products, and edible biofilms, in addition to studies validating its potential in health promotion and disease prevention, highlighting gaps and opportunities for future research.

## 2. Material and Methods

### 2.1. Protocol and Registration

A scoping review was conducted according to the Preferred Reporting Items for Systematic Reviews and Meta-Analyses Extension for Scoping Reviews (PRISMA-ScR) and registered on the Open Science Framework (https://osf.io/gv7wz, accessed on 5 August 2025). The PRISMA-ScR checklist is available as Supplementary Material.

### 2.2. Search Strategy

The literature search strategy was developed using the PCC acronym, in which P (population) was defined as plantain peel, C (concept) as composition and functionality of plantain peel, studies validating its potential in promoting health and preventing disease, its use for the development of new food products, microbiological activity, and enzyme production, and C (context) global. The guiding research question was: What evidence exists regarding the composition, functionalities, and potential applications of plantain peel (*Musa paradisiaca*) in food, health, and biotechnology?

Searches were conducted in the PubMed, Embase, Web of Science, Scopus, and Google Scholar databases from their inception until December 2025, without language restriction. Search strategies were developed using MeSH terms and adapted to each database. The following strategy was applied: “plantain” AND “*Musa paradisiaca*”, “flour” AND “*Musa paradisiaca*”, “plantain” AND “*Musa paradisiaca*” AND “peel”, “banana peel” AND “*Musa paradisiaca*”, “bioactive compounds” AND “plantain” AND “*Musa paradisiaca*”, “plantain” AND “*Musa paradisiaca*” AND “nutritional”, “plantain peel” AND “food”, “plantain peel” AND “food functionality”, (“plantain” OR “*Musa paradisiaca*”) AND (“nutritional impact” OR “nutritional properties”), “industrial applications” AND “banana peel. The reference lists of included articles were also manually searched. References were managed using a reference management system [13].

### 2.3. Eligibility Criteria, Study Selection, and Data Extraction

Two independent authors (APP and CP) selected the studies using a two-phase process. In phase one, the authors independently evaluated the titles and abstracts and applied the eligibility criteria. In phase two, the same two authors conducted a full-text reading of the selected material, using the same eligibility criteria. Throughout both phases, any disagreements among the authors were resolved through a consensus meeting. If a consensus could not be reached, a third author (JACB) was consulted to reach a final decision.

The inclusion criteria comprised studies where (1) the sample was plantain peel (*Musa paradisiaca*), and (2) it was used for enzyme production, microbiological activity, development of new food products, and studies validating potential in promoting health and preventing disease, as well as its composition and functionality.

The studies were excluded because the samples were not plantain (*Musa paradisiaca*) and were used for biofuel production and bioremediation, topics considered outside the scope of this review.

The following data were extracted from the included studies: author, year, country of publication, objectives, methods, main results, and limitations.

## 3. Results and Discussion

### 3.1. Selection of Studies

**Figure 1:**
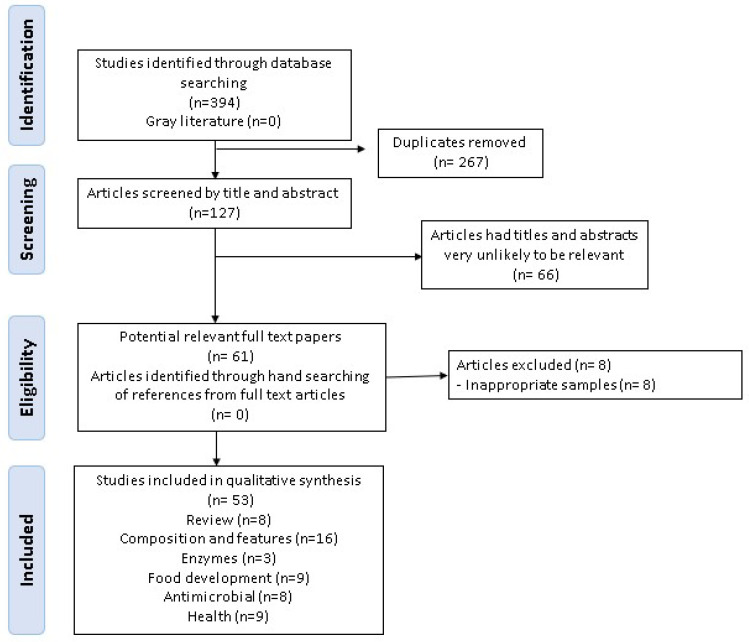
Flowchart of studies selected for this review.

After removing duplicates from the databases searched, the initial study was reduced to 127 articles. The subsequent reading of abstracts, carried out in pairs, resulted in the selection of 61 articles relevant to the topic, excluding articles addressing the use of plantain peel for the production of biofuels and bioremediation. Subsequently, eight articles were excluded from this study because they did not present the selected sample, i.e., plantain peel. The remaining 53 articles were read in full and subsequently categorized according to their primary focus as follows: 16 studies on the composition and functionalities of plantain peel, elucidating its functionality attributed to bioactive compounds extracted from green and/or ripe plantain peel; 3 studies on enzyme production; 9 articles on the development of food products or edible biofilms; 9 studies addressing the use of plantain peel in the health field, pointing out the therapeutic potential of the peel and suggesting its application in the prevention and treatment of certain diseases; 8 studies focused on antimicrobial action; and 8 review articles that complement this research by analyzing plantain production and consumption data (Figure 1).

Eight studies were conducted in Indonesia, contributing to Asia’s prominence as the region with the highest number of published articles (*n* = 18). The next geographical area to be examined was South and Central America, which includes thirteen countries, including Colombia, Panama, Brazil, Venezuela, Ecuador, Puerto Rico, and Mexico. The African continent presented ten studies on the by-product in Nigeria, Ghana, and South Africa. Finally, only three articles originated from Europe. Notably, no studies in this area were present from North America and Australia.

A global overview of research on plantain peel reveals a markedly polarized geographical distribution, with most studies concentrated in tropical regions where the fruit is widely cultivated. This concentration of research in production nations suggests that the primary motivation for the studies is the local need to manage abundant agro-industrial by-products and align them with the principles of the circular bioeconomy.

### 3.2. Composition and Functionality of Plantain Peel

Table 1 presents the results of studies evaluating the proximate composition of plantain peel under different ripening conditions and processing methods. Pilco et al. [14] analyzed the ripe raw peel, observing low protein and lipid content, with a significant amount of dietary fiber and ash. Similarly, Oyeyinka and Afolayan [15] investigated flour obtained from ripe peel, and reported the increased levels of carbohydrates, proteins, and dietary fiber, possibly due to the concentration of nutrients resulting from dehydration. On the other hand, Pham et al. [16] studied the flour from unripe plantain, finding higher protein and lipid values when compared to flour from ripe peel, as well as high ash content, suggesting that the stage of ripeness directly influences nutrient retention and availability. Similar results were reported by Nasrin et al. [17] for green plantain peel, which showed significant levels of dietary fiber and minerals. In addition, these studies show plantain peel is a nutritionally rich by-product with potential for technological application in foods, especially as an alternative source of fiber, minerals, and functional compounds.

The data on the proximal composition and minerals of plantain peel are presented in Table 1.

Table 1 shows that the proximate composition of plantain peel varies according to the stage of ripeness and the type of processing. Green peel flour has the highest levels of protein, carbohydrates, dietary fiber, and fixed mineral residue. In contrast, fresh ripe peels have lower protein and fiber values. The lipid content, although low in all samples, also increases after processing, reaching 14.26 g/100 g in green peel flour. These results indicate that green plantain peel is a promising source of nutrients and dietary fiber. These increases are associated with the concentration of nutrients resulting from dehydration and the preservation of structural compounds typical of immature fruits, such as cellulose, hemicellulose, and lignin, reflecting the partial degradation of structural components during ripening and the relative increase in moisture [14,15,16,17].

Pectin, with a yield of 12.43% from dry peel subjected to enzymatic extraction, was reported by Xie et al. (2023) [18]. The fiber content is also significant: cellulose (19.6–33.9%), hemicellulose (up to 45.6%), and lignin (14.3–16.8%), as reported by Emaga et al. [19]. The presence of starch was confirmed, especially in green peel with high moisture content (87.16%), as reported by Hernandez-Carmona [20]. In peel flours, moisture content was reported to be around 10.42% [21].

The peel’s structural composition is characterized by an exceptionally high dietary fiber content, with a strong predominance of the insoluble fraction, including cellulose, hemicellulose, and a significant proportion of lignin. This specific profile is key to its functional properties: the lignin, peel flour’s chemical inertness and thermal stability. Furthermore, the high concentration of insoluble fiber and the resulting increased intestinal viscosity are associated with the protective effect observed against systemic pathogen invasion (e.g., *Salmonella* in in vivo studies), suggesting a crucial physical barrier effect in the gastrointestinal tract. In eight studies, plantain peel was characterized as a matrix rich in functional compounds [12,14,16,18,22,23,24]. High levels of dietary fiber, such as pectin, and polyphenols, including gallic acid, rutin, and quercetin, are noteworthy and are associated with high antioxidant capacity, as evidenced by different analytical methods. These data are presented in Table 2.

Regarding bioactive compounds, different classes were identified. Fatty acids and sterols were detected in the extracts in analyses performed by Pilco et al. [14].

Antioxidant activity was confirmed by two studies: Sriarumtias et al. [22] evaluated the antioxidant activity of the methanolic extract of plantain peel. This result indicates moderate antioxidant capacity, observed in the context of microemulsion development for cosmetic application, which may have influenced the processing of the extract and its antioxidant efficacy. However, Padilla-Camberos et al. [23] also evaluated the antioxidant activity suggesting greater antioxidant capacity. This efficacy is likely due to the presence of multiple hydroxyl groups on the aromatic rings of these compounds, which allows them to efficiently donate hydrogen atoms to neutralize free radicals and stabilize the resulting radicals, thereby terminating chain reactions [26].

The remarkable antioxidant capacity reported across multiple studies, often exceeding 80% scavenging activity in DPPH assays, is directly correlated with the peel’s high content of phenolic acids (such as ferulic and gallic acids) and potent flavonoids (such as quercetin and naringenin). The diversity and quantity of flavonoids reinforce the functional potential of the bark as a nutraceutical and antioxidant ingredient [16,24].

### 3.3. Health Benefits

Table 3 presents the effects of plantain peel tested for health prevention and treatment. Almost all (n = 7) of these studies used aqueous or alcoholic extracts for testing in Wistar rats under different health treatments [27,28,29,30,31,32,33]. The only study involving humans was of mosquito repellents [34]. An African in vitro study investigated the effect of plantain peel on cardiovascular protection with angiotensin-converting enzyme (ACE) [35].

### 3.4. Applications of Plantain Peel in Food Production

The use of plantain peel has been extensively studied, focusing on its potential as an ingredient to enrich the nutritional and functional value of tenderizers, as well as for the production of edible films. Studies on the use of plantain peel in product development are compiled in Table 4.

Adding plantain peel flour to bread and cookies has shown positive effects on their nutritional composition [36,40,42]. Akhter, Al-Amin, Hossain and Kamal [40] observed that the incorporation of up to 5% plantain peel flour into bread resulted in a decrease in moisture content, accompanied by a substantial increase in ash, lipid, protein, and fiber content, along with a reduction in total carbohydrate content. Concurrently, there was an augmentation in the levels of total phenolic compounds (28.46 to 42.38 mg GAE/100 g), flavonoids (6.63 to 9.46 mg QE/g), and antioxidant activity (18.84% to 53.03%). A sensory evaluation demonstrated that bread containing up to 5% BPP exhibited sensory properties comparable to the control.

The incorporation of plantain peel flour (PPF) into food matrices, such as bakery products and pasta, reveals a consistent trade-off between nutritional enhancement and sensory stability. In cookies and snacks, studies indicate that, while PPF significantly boosts fiber, minerals (potassium), and antioxidant activity [36,42], there is a threshold for consumer acceptance, typically ranging between 10% and 20% [39]. This suggests that the starch–fiber complex in plantain peel not only alters the nutritional profile but also fundamentally changes the rheological properties of the dough. For instance, the increase in resistant starch and the reduction in oil absorption observed in noodles [39] and laminated pasta [41] demonstrate that PPF can function as a techno-functional ingredient, improving cooking quality and slowing carbohydrate digestion—a critical factor for low-glycemic index food development. In the field of fermented dairy products, the interaction between plantain peel and probiotic cultures suggests a synergistic prebiotic effect. The presence of fructooligosaccharides (FOSs) and dietary fibers in the peel appears to stimulate the viability of lactic acid bacteria, as evidenced in both goat milk kefir [45] and cow milk formulations [44]. Analysis of these studies reveals that the peel does more than just enrich the medium with phenolic compounds; it creates a protective matrix that maintains microbial counts (above 9 log CFU/mL) and enhances the accessibility of antioxidants during simulated digestion [44]. However, the sensory challenge persists: concentrations above 10% often lead to alterations in color and texture that may limit commercial scalability, despite the high acceptability index (up to 82.5%) for lower concentrations. Beyond direct consumption, the high amylose and starch content (approx. 40%) of the peel allows for its application in sustainable packaging and food processing. The development of edible films with low water vapor permeability [37] and the use of peel ash as a tenderizing agent [43] expand the scope of plantain peel from a mere nutritional additive to a versatile raw material in the circular bioeconomy. Collectively, these findings highlight that, while the functional potential is vast, the industrial application of plantain peel requires a precise balance between its bioactive load and its impact on the physical–sensory structure of the final product.

The main limitation to using plantain peel as an ingredient in food production lies in the consumer sensory acceptance. High concentrations of plantain peel flour can negatively affect sensory acceptance, leading to undesirable flavors (such as bitterness), compromised textures (soft, crumbly, or coarse), and, in general, lower overall consumer appeal. Studies have shown that exceeding 5% in bread, 20% in fermented milk, or 60% in cookies can reduce product acceptability. This is attributed to the high fiber content, which can result in a rougher texture, and the presence of phenolic compounds which contribute to astringent or bitter notes [36,40,42,44]. Furthermore, the absence of gluten in plantain peel impairs the gluten network when used as a wheat flour substitute, leading to decreased structural integrity and altered shapes in pastry products [40].

### 3.5. Antimicrobial Potential and Biotechnological Applications

Plantain peel has shown significant antimicrobial potential (Table 5). The significant intrinsic antimicrobial potency demonstrated by peel extracts is highly attributable to the concentration of ethanol-soluble polyphenols, including tannins and specific saponins, which act as membrane-active antimicrobial agents. The primary mechanism involves the disorganization of microbial cell membrane integrity and the inhibition of essential enzymatic activity, due to the ability of these phenolic compounds to bind to and precipitate proteins in the cell wall and cytoplasm [46,47].

Antimicrobial activity is attributed to bioactive compounds in the peel, including flavonoids, tannins, alkaloids, phenols, saponins, and terpenoids [11]. Kapadia et al. [51] demonstrated that the alcoholic extract of plantain peel showed significant inhibition against periodontopathogenic bacteria *Porphyromonas gingivalis* and *Aggregatibacter actinomycetemcomitans*. Using agar diffusion and broth dilution methods, the authors observed the complete inhibition of microbial growth at a concentration of 60 mg/mL, where the minimum inhibitory concentration (MIC) was determined by reduction efficacy.

Beyond its direct biological activity, plantain peel serves as a valuable resource in green nanotechnology. It acts as a cost-effective and eco-friendly reducing and stabilizing agent for the biosynthesis of metallic nanoparticles, which function as the primary antimicrobial agents.

A study of Wulandari et al. [53] used Kepok plantain peel extract to synthesize silver nanoparticles, which were coated with chitosan to formulate a hand sanitizer gel. Chitosan-coated silver nanoparticles exhibited antibacterial activity against *Staphylococcus aureus* and *Escherichia coli*. The gel had a greater impact on *S. aureus* than *E. coli*, and chitosan demonstrated a synergistic effect on the antimicrobial activity of the nanoparticles. Pham, Nguyen, Pham, Pham and Tran [16] used aqueous extract from plantain peel for the biosynthesis of silver nanoparticles (nAgs). The biosynthesized nAgs demonstrated antimicrobial action against *Staphylococcus aureus* (Gram-positive) and *Escherichia coli* (Gram-negative). The inhibition zones were 14.1 ± 0.1 mm for *S. aureus* and 11.4 ± 0.1 mm for *E. coli*, with a minimum inhibitory concentration (MIC) of 13.5 μg/mL for both.

The significant intrinsic antimicrobial potency was largely attributable to the concentration of ethanol-soluble polyphenols, including tannins and specific saponins, which act as membrane-active antimicrobial agents. The primary mechanism involves the disorganization of microbial cell membrane integrity and the inhibition of essential enzymatic activity, due to the ability of these phenolic compounds to bind to and precipitate proteins in the cell wall and cytoplasm.

The versatility of plantain peel extends to other metallic composites as well. Innovative applications also extend to photocatalytic nanocomposites (e.g., TiO_2_/rGO), where the antimicrobial mechanism is driven by the generation of reactive oxygen species (ROS) under solar radiation [11]. This transition from simple biological extracts to complex metallic composites highlights the versatility of plantain peel in the circular bioeconomy, moving beyond traditional food applications toward high-tech antimicrobial solutions such as hand sanitizer gels and self-disinfecting surfaces.

### 3.6. Application of Plantain Peel in Enzyme Production

This scoping review identified three international studies (Nigeria, Panama, India) demonstrating the promising potential of bioconverting plantain peels, an abundant and underutilized agro-industrial by-product in tropical regions, into a substrate for the production of industrially relevant enzymes. The literature suggests that, particularly, pectinase biosynthesis results in improved fermentation parameters and increased enzyme yield. Pectin-rich agro-wastes enhance fermentation by acting as strong inducers and nutrient-rich and physically favorable substrates, and by allowing the statistical optimization of the medium and conditions, which together increase biomass, specific productivity, and overall pectinase yield while reducing cost. Thus, the use of plantain peel represents a cost-effective, readily available feedstock for industrial biotechnology, while simultaneously reducing the environmental burden associated with agricultural waste management [54].

According to Adeniran et al. [55] and Sethi, Nanda and Sahoo [54], plantain peel stands out as a promising raw material for the production of industrial enzymes such as pectinase, beta-amylase, and amyloglucosidase (Table 6). This potential is mainly due to its favorable chemical composition, with high carbohydrate and starch content (623 g/kg total carbohydrates and 374 g/kg starch), which provide carbon and energy for microbial growth and enzyme biosynthesis. The high protein content (91 g/kg) is also essential for cell metabolism and enzyme development. The pectin presence in the peel (10–21%) makes this substrate particularly suitable for pectinase production [56].

This residue also contains several essential micronutrients including N, P, K, Ca, Mg, Fe, Cu, Mn, Zn, and Na, which play an important role as enzymatic cofactors and in microbial cell metabolism. Another advantage is its low fat and crude fiber content (8.7%), which facilitates enzyme extraction and purification, often reducing the need for pretreatment [18].

In Nigeria, plantain peel has been exploited in the production of beta-amylase and amyloglucosidase. Although cassava peel and spent brewing grains have shown good results for amyloglucosidase production, peels remain a viable, accessible, and sustainable alternative [55].

For the production of enzymes, according to Monrroy, Rueda, Aparicio and García [10], plantain peel emerges as a viable and promising substrate for the production of citric acid (CA) via solid-state fermentation with *Aspergillus niger*. The viability of this bioprocess is directly attributable to the advantageous physicochemical composition of the plantain peel, specifically its high concentration of fermentable nutrients: it contains a substantial total carbohydrate content of 623 g/kg and a starch content of 374 g/kg, complemented by a protein fraction of 91 g/kg. This rich profile effectively provides the necessary carbon and nitrogen sources required to support optimal microbial proliferation and the subsequent biosynthesis of citric acid (CA). This process also benefits from the fact that it provides a way to value an abundant and underutilized agro-industrial by-product that would otherwise contribute to environmental waste. Furthermore, the implementation of solid-state fermentation (SSF), employed in this study, demonstrated effective performance. SSF offers a distinct advantage over submerged fermentation (SmF), potentially mitigating the negative inhibitory effects associated with the dissolution of trace elements (microelements) into the liquid medium, as commonly observed in SmF (Table 4). However, a significant limitation to consider is that, despite the high macronutrient content of raw plantain peel, the effective concentration of these compounds within the actual fermentation mixture might be reduced, thereby requiring mandatory substrate supplementation. Although this study did not report major process limitations, the optimization of critical parameters, such as pH and temperature, remains paramount. Specifically, maintaining stringent control over the pH, ideally sustaining an acidic condition (pH < 2), is essential to effectively inhibit the co-production of undesirable organic acids and minimize the risk of microbial contamination [10].

### 3.7. Contribution/Challenges/Future Perspectives

According to the studies evaluated in this scoping review, there are several challenges in the use of plantain peel, mainly related to its use as a raw material for value-added products or for specific purposes, such as the production of antimicrobials and/or enzymes. One of the challenges reported is the variability in composition and characteristics of the peel, which can vary significantly depending on the stage of fruit ripeness, the cultivar, and pre-processing conditions (e.g., drying and grinding) [50,54,55]. This variability affects the efficiency of extraction processes and the quality of products such as nanoparticles or enzymes. For example, the size of biosynthesized silver nanoparticles can be affected by variations in temperature and pH during synthesis and drying [50].

A critical aspect requiring rigorous attention is the mandatory optimization of bioprocess conditions. Numerous investigations underscore the complexity involved in optimizing key variables, such as incubation time, temperature, pH, and inoculum concentration, particularly due to the intricate interactions among these factors [16,50,53]. Furthermore, the complex composition of the plantain peel matrix introduces potential interferences. For instance, the presence of specific compounds (AgCl) during silver nanoparticle synthesis compromises the purity of the target product [16]. An additional concern pertains to the biosafety risks associated with certain fermenting microorganisms, specifically the potential for strains such as Aspergillus flavus and Aspergillus fumigatus to produce aflatoxins [55]. Finally, it must be acknowledged that most of the current research is confined to the laboratory scale. The successful upscaling and transition to industrial production face technical and biosafety challenges [7,25,34,37,40,57].

The analyzed studies consistently point toward highly promising prospects for the comprehensive valorization of plantain peel and the development of novel applications. One area with substantial potential is diversifying high-value products. The peel can serve as an effective substrate for the biosynthesis of a range of enzymes, including pectinase, β-amylase, and amyloglucosidase, as well as for the production of citric acid and nanofibers suitable for the development of food films [9,10,54,55]. Furthermore, its utility extends to the green synthesis of nanomaterials, such as silver nanoparticles (AgNPs) and zinc oxide nanoparticles (ZnONPs) [48,50,53]. Consequently, future research efforts should be directed toward optimizing synthesis conditions to gain meticulous control over the size, shape, and dispersion of these nanoparticles, thereby enhancing their antimicrobial and catalytic properties. These nanomaterials hold increasing significance for applications across various sectors, including healthcare (such as disinfectants and antibacterial agents), water purification, and advanced food preservation techniques [11].

An area of significant promise lies in exploring the therapeutic potential of the bioactive compounds inherent in plantain peel, such as flavonoids, tannins, and alkaloids. There is substantial potential for developing novel antimicrobial agents derived from these phytochemicals [9,51,54]. Furthermore, research should prioritize the rigorous identification and isolation of new molecules with confirmed pharmacological potential, advancing the use of this agro-waste in pharmaceutical and health-related fields.

Another promising field of study is the use of plantain peel as an ingredient in the formulation of new foods rich in bioactive compounds, thereby broadening its range of applications. The circular bioeconomy, which aims to utilize agro-industrial waste such as plantain peel, is a growing trend with great potential in the future.

While this review successfully maps the rich nutritional and functional composition of plantain peel, a crucial area demanding further scientific rigor is the comprehensive assessment of its safety profile, particularly concerning antinutrients and contaminants. Agro-industrial waste materials, including fruit peels, can accumulate high levels of compounds such as oxalates, phytates, and tannins, which may impair mineral absorption and protein digestibility. Moreover, residual pesticide accumulation in the peel poses a significant toxicological concern that is currently underreported in the literature. Therefore, mandatory future studies must focus on: (1) quantifying the specific levels of major antinutrients in various peel preparations and (2) investigating the efficacy of common food processing techniques (e.g., fermentation, cooking, drying) in significantly reducing these compounds and residual pesticides to levels safe for human and animal consumption, thus enabling safe commercial application in products like functional flours and additives.

A critical requirement for advancing the commercial and scientific application of plantain peel is the establishment of Standardized Extraction Protocols (SEPs). Currently, a significant challenge, and a source of high compositional variability, stems from the lack of uniformity in laboratory methods. Studies often employ disparate solvent systems (e.g., methanol, ethanol, ethyl acetate, or water) at varying temperatures, times, and solvent-to-solid ratios, which results in incomparable data on yield, purity, and bioactivity. Therefore, future research must shift focus from simply proving efficacy to method standardization. This involves comparative studies to determine the single, most efficient, scalable, and environmentally friendly solvent/protocol that maximizes the extraction of target bioactive compounds while ensuring reproducibility across different processing facilities and geographical origins.

## 4. Final Considerations

Plantain peel has demonstrated potential as an agro-industrial waste product, aligned with the principles of sustainability, the circular economy, and technological innovation. Its composition of fibers and starch, as well as bioactive compounds such as phenolics and carotenoids, gives it significant functional properties, including antioxidant and antimicrobial activities.

The applicability of plantain peel in the food industry is evidenced by its incorporation into various formulations, including breads, cookies, pasta, jams, and fermented products, thereby enriching their nutritional and functional profiles. In addition, its potential extends to the production of edible films and its use as a meat tenderizer. However, the sensory acceptability of food products, containing the peel, can be improved through the optimization of formulations and processes.

Despite significant advances, the transition from laboratory scale to industrial production still faces hurdles, including variability in peel composition, the need for optimized process conditions, and interference from co-extracted compounds. To solidify its industrial viability, future research must prioritize standardizing compound extraction protocols and further elucidating the mechanisms of action of its bioactive molecules. Crucially, addressing the existing knowledge gaps regarding the toxicological profile of the peel is mandatory. Specifically, future studies should focus on quantifying and optimizing processing methods to reduce key antinutrients (e.g., oxalates, phytates) and pesticide residues. Overcoming these safety and processing challenges is essential for translating the peel’s laboratory-proven functionality into safe, viable industrial food applications, thereby maximizing the use of this agricultural waste, and contributing effectively to the circular bioeconomy, food security, and human health.

## Figures and Tables

**Table 1:** Proximal composition and minerals of plantain peel.

Components	Plantain Peel			
	Pilco et al. [14]	Oyeyinka and Afolayan [15]	Pham et al. [16]	Nasrin et al. [17]
Ripening stage	Ripe fresh	Ripe flour	Unripe fresh	Unripe flour
Protein (g/100 g)	0.74	2.23	10.17	4.16
Carbohydrate (g/100 g)	8.3.	68.57	62.91	72.52
Lipid (g/100 g)	0.47	1.1	14.26	6.02
Dietary fiber (g/100 g)	0.87	4.06	-	7.68
Fixed mineral residue (g/100 g)	1.55	2.23	12.63	9.66
Potassium (mg/100 g)	83.0	-	-	-
Magnesium (mg/100 g)	-	-	6.00	-
Calcium (mg/100 g)	-	-	7.00	-

**Table 2:** Overview of studies evaluating the functional properties and potential applications of plantain peel.

Objectives	Method Used	Main Results	Limitations
Recovery of pectin from green plantain peels and its application in packaging films by Xie et al. [18]	Enzymatic extraction of pectin, film production, and analysis of yield and properties	Pectin yield was 12.43% (control) and 25.00% (cellulase-assisted). Films showed improved mechanical properties.	Scalability and industrial optimization were not evaluated.
Formulation of antioxidant microemulsions using Kepok plantain peel by Sriarumtias et al. [22]	Methanolic extraction; DPPH assay; microemulsion formulation and evaluation	Microemulsions containing Kepok peel extract demonstrated stability and antioxidant activity.	Antioxidant activity was lower than that of vitamin C.
Determination of proximate composition and identification of phytochemicals with antimicrobial activity by Pilco et al. [14]	Proximate analysis; sequential ethanolic extraction; fractionation; antimicrobial testing	The peel contained 11% total solids and showed nutritional potential. Ethanolic extracts exhibited antimicrobial activity.	No specific antimicrobial compounds were isolated or identified.
Evaluation of the effect of ripening and extraction conditions on polyphenols and antioxidant activity by Pham et al. [16]	Proximate analysis; variation in extraction parameters; analysis of total phenolic content and antioxidant activity (DPPH)	Yellowish-green peels and optimized extraction (50% ethanol, 1:40, 1.5 h, 60 °C) resulted in the highest phenolic content and antioxidant activity	Specific phenolic compounds were not identified.
Evaluation of the wound healing activity of plantain peel extracts by Padilla-Camberos et al. [23]	Phytochemical screening; antioxidant analysis (DPPH); toxicity testing; in vivo wound healing model (rats); histology	Peel extracts exhibited antioxidant properties and positively influenced wound-healing processes.	Specific bioactive compounds responsible for the healing effect were not isolated.
Evaluation of carotenoid concentration in pulp and peel of 15 plantain cultivars at two ripening stages by Aquino et al. [24]	HPLC-DAD analysis of individual carotenoids and spectrophotometric analysis of total carotenoids in the pulp and peel	Cultivar “Terrinha” exhibited the highest levels of α- and β-carotene in the peel. The ripening stage significantly influenced carotenoid content.	Bioavailability of the carotenoids was not evaluated.
Optimization of a facial mask formulation containing plantain peel flour and evaluation of its antioxidant activity by Apriani et al. [25]	Peel flour preparation; phytochemical screening; mask formulation; antioxidant activity (DPPH) and optimization	The optimized formulation demonstrated low antioxidant activity.	A comparison between the efficiency of flour and pure peel extract was not performed.
Investigation of phytochemicals, antioxidant and anti-inflammatory activities, and toxicity of ‘Nam Wah’ plantain peel by Widoyanti et al. [12]	Sequential extraction; phytochemical screening; antioxidant (DPPH, FRAP) and anti-inflammatory (NO scavenging, 15-LOX inhibition) assays; toxicity; molecular docking	“Nam Wah” peel showed antioxidant and anti-inflammatory potential.	Study restricted to in vitro and in silico models, and a lack of in vivo validation.

**Table 3:** Analytical summary of primary studies on the biological effects and therapeutic potential of plantain peel.

Objectives	Methods	Mains Results	Limitations
Investigate the inhibitory effects of phenolic extract from different stages of ripeness of plantain peels on ACE and their antioxidant properties in vitro by Oboh et al. [35]	Phenolic extraction of plantain peels with methanol. In vitro tests: ACE inhibition, ABTS antioxidant capacity, and reducing property (FeCl_3_). Characterization of phenolic constituents.	Dose-dependent ACE inhibition. Green peel: ↑ ACE inhibition and ↑ antioxidant activity. Ripe peel: ↓ inhibition.	In vitro study; in vivo mechanisms and bioavailability of the compounds have not been investigated.
Analyze the potential of Kepok plantain peel extract to lower triglyceride levels by Indriawati and Khalifah. [27]	Wistar rats. Peel extract (100, 200, 400 mg/kg BW) for 14 days. Maturation stage not reported.	↓ triglycerides at all doses of the extract tested.	Mechanism of action unclear.
Evaluate the antihyperglycemic effects of hydroethanolic extracts from plantain leaves and peel by Ahmed et al. [28]	Wistar rats with diabetes. Oral treatment with hydroethanolic extracts from the leaf or peel of *M. paradisiaca* (100 mg/kg/day) for 28 days. Maturation stage not reported.	The extracts improved glucose tolerance, ↑ insulin, and C-peptide. ↑ insulin receptor expression.	Single dose tested. Dose–response studies would be useful.
Investigate the potential cardioprotective effects of the aqueous extract of ripe plantain peel by Suleiman et al. [29]	Wistar rats. Pretreatment with MPPE (100, 200, 400 mg/kg) or aspirin (30 mg/kg).	MPPE (400 mg/kg) showed beneficial effects comparable or superior to aspirin.	Only one variety (ripe peel) was tested.
Determine the effect of plantain peel extract on MDA levels in rats exposed to cigarette smoke by Damarjati et al. [30]	Wistar rats. Groups: negative control; exposed to smoke; smoke with peel extract (200 mg/kg); and smoke with peel extract 400 mg/kg). Maturation stage not reported.	Plantain peel extract, at both doses, ↓ MDA levels.	The type of peel extraction was not detailed. The exact mechanism of MDA reduction has not been elucidated.
Investigate the effect of extracts from green and ripe plantain peel on the sexual behavior of rats by Oyeleye et al. [31]	Wistar rats with erectile dysfunction. Aqueous extracts of UPP and RPP (200 and 400 mg/kg).	Treatment with UPP and RPP reversed dysfunction, inhibited enzymes, and increased hormone levels.	Toxicity profile and long-term effects were not evaluated.
Development of a mosquito repellent soap using indigenous lye from the ashes of green and ripe plantain peels by Adjei et al. [34]	Production of lye from plantain peel ash. Formulation of bar soap and shower gel. Repellency testing on 40 humans.	The soap provided 3–4 h of protection against mosquito bites.	The study focuses on the ash from the peel, not on the direct properties of the peel as a health agent.
Evaluate wound healing using a nano-hydrogel infused with bioactive components from plants and plantain peel by Sofini et al. [32]	Wistar rats. Tests: antibacterial (*S. aureus*, *E. coli*), in vitro scratch wound healing assay, and in vivo burn wound healing. Maturation stage not reported.	The hydrogel showed antibacterial activity—faster healing of burn wounds compared to the compound without the nano-gel.	The exact individual contribution of plantain peel is inferred, but not isolated.
Investigate the potential anxiolytic effect of green banana peel ethanol extract (BPE) by Soeliono et al. [33]	Wistar rats. Groups: CMC control, alprazolam (0.4 mg/kg), tryptophan (270 mg/kg), 5-HTP (18 mg/kg), and BPE (140, 280 mg/kg).	BPE (140 and 280 mg/kg) did not show a significant anxiolytic effect.	The observed lack of anxiolytic effect contrasts with previous findings in other varieties.

Free radical-scavenging capacity; 2,2′-Azino-bis (3-ethylbenzothiazoline-6-sulfonic acid) (ABTS), angiotensin-converting enzyme (ACE), carboxymethyl cellulose (CMC), plantain peel ethanol extract (BPE), malondialdehyde (MDA), ripe plantain peel extract (MPPE), unripe plantain peel extract (UPP) and ripe plantain peel (RPP).

**Table 4:** Utilization of plantain peel for food production.

Product Developed	Usage/Country	Sensory Analysis and Functional Compound Results	Limitations
Cookies Arun et al. [36]	Unripe cultivar Nendran plantain peel flour (NPF) (5%, 10%, and 15%). India.	NPF proved to be a rich source of dietary fiber and antioxidants. Cookies with 10% NPF showed good acceptability, whereas concentrations >10% reduced it.	Sensory acceptability decreased at concentrations >10%.
Edible films Anchundia et al. [37]	Unripe peel flour (38.11% starch) with acetylsalicylic acid (ASA). Ecuador.	The physical characteristics of the films resembled those of pure starch, except for the significantly lower water vapor permeability.	Shelf-life/storage tests were not performed.
Fermented goat milk Martharini and Indratiningsih [38]	Ripe plantain peel flour—Kepok (0%, 1%, and 2%). Indonesia.	Kefir quality complied with Codex standard 234–2003 across all treatments. The optimal formulation contained 3% *L. acidophilus* and 1% plantain peel flour.	Other flour concentrations and milk types were not evaluated.
Instant noodles Aji et al. [39]	Ripe Kepok plantain peel flour (KPF) (10%, 20%, and 30%). Indonesia.	Substituting 30% of starch with KPF improved gelatinization properties and overall quality, demonstrating its potential for nutritious, sustainable noodles.	Limited nutritional and sensory evaluation.
Enriched whole wheat bread Akhter et al. [40]	Ripe plantain peel flour (5%, 7%, and 10%). Bangladesh.	Flour incorporation significantly altered bread characteristics. Enrichment with up to 5% plantain flour maintained sensory acceptability comparable to the control.	Sensory acceptability was not evaluated for higher concentrations.
Gluten-Free Rolled Pasta Bello-Pérez et al. [41]	Whole unripe plantain flour (WUPF) and unripe plantain pulp flour (UPF). Mexico.	WUPF produced gluten-free dough with reduced starch hydrolysis and increased dietary fiber content.	Sensory evaluation was not performed.
Biscuits Paramitasari et al. [42]	Banana and plantain peel flour (BPF-J—Janten and BPF-K—Kepok) (20%, 40%, and 60%). Ripeness stage 4–5 (Von Loesecke scale). Indonesia.	The incorporation of BPF-J and BPF-K improved both the nutritional profile and functional properties of the biscuits.	Application limited to biscuits; other products were not tested.
Meat tenderizer Diaba et al. [43]	Ashes from unripe plantain peels (UPPA) and their filtrate (UPPAF). Ghana.	Green plantain peel ashes, and their filtrate, contained elements within FAO/WHO limits and proved effective as meat tenderizers.	Potential limitations in sample size or marination process.
Fermented milk Silva et al. [44]	Fresh unripe plantain peel (5, 10, and 20%). Brazil.	Digestion simulation indicated high bioaccessibility of bioactive compounds, suggesting effective absorption.	High concentrations negatively affected sensory acceptance.

**Table 5:** Use of plantain peel as an antimicrobial agent.

Product Obtained from Plantain Peel Extract	Microorganism(s) Tested	Evaluation Method	Microbial Efficacy	Limitations
Zinc oxide nanoparticles (ZnONPs) Imade et al. [48]	*Salmonella enterica*, *Klebsiella pneumoniae*, *Bacillus cereus* MTCC 430, *Staphylococcus aureus* 26923	Diffusion and dilution in broth and nutrient agar	Green-synthesized ZnONPs demonstrated significant antibacterial efficacy.	N/A
Silver nanoparticles (AgNPs) Pham et al. [16]	*Staphylococcus aureus* ATCC 29213 and *Escherichia coli* ATCC 25922	Inhibition zone and minimum inhibitory concentration	Demonstrated antimicrobial activity against *S. aureus* and *E. coli*	Influence of coating biomolecules on surface properties
AgNPs coated with chitosan Widoyanti et al. [12]	*Staphylococcus aureus* and *Escherichia coli*	Diffusion with nutrient broth and nutrient agar.	Shows promise for antiseptic gel formulation	Stability testing was limited to a short duration (140 h).
Gold nanoparticles (AuNPs) Vijayakumar et al. [49]	Biofilm (*Enterococcus faecalis*), cytotoxicity (A549 cells), ecotoxicity (*Ceriodaphnia cornuta*)	Inhibition and cytotoxicity tests	Effective inhibition of biofilm formation by *E. faecalis* (100 mg/mL).	The study focused on synthesized AuNPs; the effect of the isolated extract was not evaluated.
AgNPs biosynthesized (BPAgNPs) Kifle et al. [50]	*Bacillus cereus*, *Staphylococcus aureus*, *Escherichia coli*, *Morganella morganii*, *Aspergillus niger*, *Alternaria alternata*, *Penicillium digitatum*, *Fusarium oxysporum*	Diffusion on disk (bacteria) and well (fungi)	Potent antibacterial and antifungal activity; applicable in reducing post-harvest losses	Long-term toxicity was not addressed.
Alcoholic extract of plantain peel Kapadia et al. [51]	*Porphyromonas gingivalis* and *Aggregatibacter actinomycetemcomitans*	Diffusion and serial dilution in broth	Antimicrobial activity against *P. gingivalis* and *A. actinomycetemcomitans*	In vitro study only; comparison of varieties or methods was absent.
TiO_2_/reduced graphene oxide nanocomposite (rGO) Utami et al. [11]	*Escherichia coli* and *Staphylococcus aureus*	Disk photocatalysis under sunlight	Achieved efficient and sustainable bacterial inactivation under sunlight	Industrial-scale feasibility and cost-effectiveness were not explored.
Feed with non-starch polysaccharides (NSP) Parsons et al. [52]	*Salmonella gallinarum*	In vitro test with B1OXI+ cells and in vivo test in chickens	Reduced *S. gallinarum* invasion in the liver and spleen, despite increased cecal load	Increased bacterial load in the cecum

**Table 6:** Bioconversion of plantain peel for enzyme and citric acid production.

Enzyme	Raw Material	Method	Main Results	Limitations
Citric acid (CA) Monrroy et al. [10]	Unripe plantain peel	Solid-state fermentation (SSF) with *Aspergillus niger* (30 °C, pH 1.4, 10% substrate consistency)	The maximum CA production of 29 g/kg on a dry matter basis was observed on the fourth day (96 h). pH was the most important parameter.	Strict pH control is required to achieve optimal yields.
Beta-amylase and amyloglycosidase Adeniran et al. [55]	Unripe plantain peels, cassava peels, yam peels, plantain peels, and spent brewery grains (SBGs)	Solid-state fermentation (SSF) and static submerged fermentation using *Aspergillus niger* (room temperature, 6 days)	*Aspergillus niger* produced the highest level of beta-amylase (33.2 EU) in plantain peel medium under the solid-state cultivation method. Plantain peels favored the production of beta-amylase.	Tested strains of *A. flavus* and *A. fumigatus* produced toxins, limiting their potential compared to *A. niger*.
Pectinase Sethi et al. [54]	Unripe plantain peel	SSF and liquid static surface fermentation (LSSF) with *Aspergillus terreus* NCFT 4269.10 (30 °C, 96 h, pH 5.0)	Plantain peels were the most suitable for pectinase biosynthesis (LSSF: 550 +/− 70.71 U/mL; SSF: 6500 +/− 1116.21 U/g).	The study primarily used an optimization method of one variable at a time (OVAT), which may not fully capture the complex interactions between parameters.

## Data Availability

No new data were created or analyzed in this study. Data sharing is not applicable to this article.

## Supplementary Materials

The following supporting information can be downloaded at: https://www.mdpi.com/article/10.3390/foods15071133/s1, S1: Preferred Reporting Items for Systematic reviews and Meta-Analyses extension for Scoping Reviews (PRISMA-ScR) Checklist [58].
